# Postnatal developmental trajectory of sex-biased gene expression in the mouse pituitary gland

**DOI:** 10.1186/s13293-022-00467-7

**Published:** 2022-10-11

**Authors:** Huayun Hou, Cadia Chan, Kyoko E. Yuki, Dustin Sokolowski, Anna Roy, Rihao Qu, Liis Uusküla-Reimand, Mariela Faykoo-Martinez, Matt Hudson, Christina Corre, Anna Goldenberg, Zhaolei Zhang, Mark R. Palmert, Michael D. Wilson

**Affiliations:** 1 Genetics and Genome Biology, SickKids Research Institute, Toronto, ON Canada; 2 Department of Molecular Genetics, University of Toronto, Toronto, ON Canada; 3 Donnelly Centre for Cellular and Biomolecular Research, Toronto, ON Canada; 4 Interdepartmental Program of Computational Biology and Bioinformatics, Yale University, New Haven, CT USA; 5 Department of Pathology, Yale School of Medicine, New Haven, CT USA; 6 Department of Cell and Systems Biology, University of Toronto, Toronto, ON Canada; 7 Department of Computer Science, University of Toronto, Toronto, ON Canada; 8 Division of Endocrinology, The Hospital for Sick Children, Toronto, ON Canada; 9 Institute of Medical Science, University of Toronto, Toronto, ON Canada; 10 Departments of Pediatrics and Physiology, University of Toronto, Toronto, ON Canada

## Abstract

**Background:**

The pituitary gland regulates essential physiological processes such as growth, pubertal onset, stress response, metabolism, reproduction, and lactation. While sex biases in these functions and hormone production have been described, the underlying identity, temporal deployment, and cell-type specificity of sex-biased pituitary gene regulatory networks are not fully understood.

**Methods:**

To capture sex differences in pituitary gene regulation dynamics during postnatal development, we performed 3’ untranslated region sequencing and small RNA sequencing to ascertain gene and microRNA expression, respectively, across five postnatal ages (postnatal days 12, 22, 27, 32, 37) that span the pubertal transition in female and male C57BL/6J mouse pituitaries (*n* = 5–6 biological replicates for each sex at each age).

**Results:**

We observed over 900 instances of sex-biased gene expression and 17 sex-biased microRNAs, with the majority of sex differences occurring with puberty. Using miRNA–gene target interaction databases, we identified 18 sex-biased genes that were putative targets of 5 sex-biased microRNAs. In addition, by combining our bulk RNA-seq with publicly available male and female mouse pituitary single-nuclei RNA-seq data, we obtained evidence that cell-type proportion sex differences exist prior to puberty and persist post-puberty for three major hormone-producing cell types: somatotropes, lactotropes, and gonadotropes. Finally, we identified sex-biased genes in these three pituitary cell types after accounting for cell-type proportion differences between sexes.

**Conclusion:**

Our study reveals the identity and postnatal developmental trajectory of sex-biased gene expression in the mouse pituitary. This work also highlights the importance of considering sex biases in cell-type composition when understanding sex differences in the processes regulated by the pituitary gland.

**Supplementary Information:**

The online version contains supplementary material available at 10.1186/s13293-022-00467-7.

## Highlights

- Male and female mouse pituitary gland gene and miRNA expression was profiled across five postnatal ages spanning pubertal development.
- Sex differences in pituitary gene expression exist prior to puberty and become more prominent upon puberty.
- Combining expression data from genes and miRNAs revealed 18 putative sex-biased gene targets of 5 sex-biased miRNAs.
- Sex differences in the proportions of somatotropes, lactotropes, and gonadotropes are predicted to occur prior to puberty.

**Supplementary Information:**

The online version contains supplementary material available at 10.1186/s13293-022-00467-7.

## Background

The pituitary gland plays a central role in regulating growth, lactation, reproduction, metabolism, stress responses, and puberty. These physiological processes are mediated by hormones released from five main anterior pituitary cell-types: growth hormone (GH) from somatotropes, prolactin (PRL) from lactotropes, follicle-stimulating hormone (FSH) and luteinizing hormone (LH) from gonadotropes, thyroid-stimulating hormone (TSH) from thyrotropes, and adrenocorticotrophic hormone (ACTH) from corticotropes [1]. Unlike the more glandular anterior pituitary, the more neural posterior pituitary consists primarily of astroglial-like pituicytes as well as axonal projections from the hypothalamus which store and release oxytocin and vasopressin into the systemic circulation [2, 3]. Non-hormone producing pituitary cells, including stem cells, and folliculostellate cells, are also present in the pituitary gland and support hormone-producing cells by functioning as progenitor cells and facilitating intercellular signaling within the pituitary [4, 5].

Both pituitary hormone production and many physiological processes regulated by the pituitary gland are sex-biased. For example, GH is secreted in a sexually different pattern in rodents—more pulsatile in males compared to females, and regulates sex-biased gene expression in liver [6]. Moreover, the clinical presentation and prevalence of pituitary-related disorders can also differ between sexes. For example, the prevalence of prolactinoma is significantly higher in women, but men are more likely to present with macroadenomas [7, 8]. While the sex differences in pituitary function and disease are well known, the gene regulatory networks underlying such differences remain elusive.

Several studies in rodents and humans have clearly highlighted sex differences in pituitary gland gene regulation. These studies include: targeted qPCR profiling of genes encoding for the main pituitary hormones in rat anterior pituitaries [9], serial analysis of gene expression (SAGE) in whole adult mouse pituitaries [10], and RNA-sequencing of adult human pituitaries as part of the Genotype-Tissue Expression (GTEx) project [11–13]. Most recently single-cell RNA-seq (scRNA-seq) has been performed in male and female adult mouse and rat pituitary glands revealing genes with sex-biased expression within specific cell types [14–16]. While most gene expression studies have focused on the adult pituitary, sex differences in pre-pubertal gene expression have been revealed using qPCR in mouse and rat pituitary glands [9, 17] and by RNA-seq in juvenile mouse gonadotropes [18]. While these studies suggest that some sex differences in pituitary gene regulation are established prior to puberty, we still lack a comprehensive view of pituitary gene regulation during postnatal development.

Another essential aspect of gene regulation is post-transcriptional regulation by microRNAs (miRNAs). While miRNA expression has been explored in pituitary glands of several mammalian species, these studies were not focused on postnatal development in males and females [19–24]. Clear examples of miRNA regulation of sex-biased gene expression have been reported in the neonatal hypothalamus and pubertal liver [25, 26], but evidence is currently limited in the pituitary gland. In one example, gonadotrope-specific *Dicer* knock-out (KO) in the mouse results in male-specific loss of follicle-stimulating hormone β-subunit (*Fshb*) gene expression, suggesting a sex-specific DICER-dependent post-transcriptional regulation of *Fshb*. This example points to the need for more comprehensive study of miRNA regulation of sex-biased gene expression in the whole pituitary.

The objective of this study was to characterize male and female pituitary gene expression during postnatal development and investigate the role of miRNAs in regulating pituitary sex differences. To achieve this, we profiled gene (3’ untranslated region sequencing, 3’UTR-seq) and miRNA (small RNA sequencing, sRNA-seq) expression in male and female mice at multiple postnatal days spanning pubertal transition to identify genes and miRNAs exhibiting known or novel sex differences. The resulting temporal gene and miRNA expression data from this study can be queried and visualized at https://wilsonlab-sickkids-uoft.shinyapps.io/pituitary_gene_mirna_shiny/. By combining this data with published single-cell RNA-seq (scRNA-seq) datasets, we provide evidence that sex differences in cell-type proportions emerge prior to the onset of puberty and likely contribute to sex biases in bulk gene expression.

## Methods

### Animal and tissue collection

All studies and procedures were approved by the Toronto Centre for Phenogenomics (TCP) Animal Care Committee (AUP 09-08-0097) (see Ethics approval for details). Conditions in which C57BL/6J mice were maintained, killed, and dissected are described previously in [17] as pituitary samples from the same animals were used in the current study.

Physical markers of puberty, vaginal opening (VO) and preputial separation (PS), were assessed every day after weaning to determine the pubertal stage of our female and male mice, respectively [27–29]. For assessing VO, the mouse was held by her base and a sterile pipette tip was used to brush the fur and assess the opening of the vagina. For assessing PS, the mouse was held on the hopper by his base and the degree of separation was assessed by gently pushing the prepuce using a sterile pipette tip [28].

Upon dissection, the pituitary gland was directly moved to RNAlater (containing 10% w/v sodium citrate tribasic dihydrate and 60% w/v ammonium sulphate) and stored at − 20 °C until RNA extraction.

### RNA extraction

Prior to RNA extraction, pituitary tissue samples were placed into bead mill tubes containing six 1.4-mm ceramic beads (MoBio Laboratories) and homogenized for 30 s at 6.5 m/s at 4 °C using an Omni Bead Ruptor 24 bead mill. RNA was extracted with the NucleoSpin® miRNA kit (Macherey Nagel) in combination with TRIzol lysis (Invitrogen) following the manufacturers’ protocols, allowing for collection of small RNA (< 200 nt) and large RNA (> 200 nt) simultaneously into separate tubes from total RNA. RNA quantity was determined using Nanodrop and the quality was assessed by an Agilent 2100 Bioanalyzer.

### Library preparation and sequencing

To construct RNA-seq libraries, we established an automated 3’UTR-seq (QuantSeq 3’mRNA-seq; Lexogen GmbH, Vienna) using the Agilent NGS Workstation (Agilent Technologies, Santa Clara) at The Centre for Applied Genomics (TCAG) (Toronto, Canada) as per the manufacturer’s protocol [30]. Briefly, 250 ng of RNA, from the large RNA fraction, was used to generate cDNA. cDNA was amplified with 17 PCR cycles as determined by qPCR analysis using the PCR Add-on kit (Lexogen). ERCC RNA spike-in Mix 1 was added following the manufacturer’s instructions. The resulting libraries were quantified with Qubit DNA HS (Thermo Fisher, Waltham). Libraries with insufficient library quantity determined by Qubit (below detection range) were excluded from further downstream processing. The remaining libraries had fragment sizes analyzed on the Agilent Bioanalyzer using the High Sensitivity DNA assay prior to sequencing. Sequencing was performed at TCAG on the HiSeq 2500 v4 flow cell (Illumina, San Diego) with SR50 bp with cycles extended to 68 bp.

Small RNA-seq libraries were generated from the small RNA fraction of the same samples (except for PD37 which was excluded from this experiment) by TCAG using NEBNext Small RNA Library Kit (New England BioLabs) according to the manufacturer’s protocol in two batches: 20 ng of RNA was used for batch 1 (replicates 1–3) and 10 ng of RNA was used for batch 2 (replicates 4–6). Ages and sexes were equally represented in the two batches. Batch effects were corrected for using RUVSeq (see Methods—mRNA and miRNA normalization and differential analysis). Sequencing was performed at TCAG on a HiSeq 2500 v4 flow cell with SR50 bp.

### mRNA sequencing reads processing

FastQC (http://www.bioinformatics.babraham.ac.uk/projects/fastqc/) was used to examine the quality of sequenced reads. Next, a customized script was used to trim both the polyAs and adapter sequences at the end of the reads. A subset of sequencing reads obtained from 3’UTR-seq show a mixture of polyAs and sequencing adapters towards the end of the reads, which are not effectively trimmed by available read-trimming tools. We developed a trimming strategy which can identify and trim off polyA sequences embedded in the adapter sequences. Only reads longer than 36 bp after trimming were used. In addition, the first 12 nucleotides were trimmed based on the manufacturer’s recommendations. After trimming, FastQC was performed again to examine read quality. At this step, we found that ribosomal reads were overrepresented through priming by oligo(dT) binding to A-rich regions in the ribosomal RNA loci. In addition, the sequence of a brain-enriched small RNA, BC1, was also overrepresented. Thus, reads that map to these overrepresented transcripts were removed. Trimmed and filtered reads were aligned to the mouse genome using a splice-aware aligner, STAR (version 2.7.0f) [31], with parameters “–outWigType wiggle –twopassMode Basic –outFilterMismatchNoverLmax 0.05”. To build the genome index used by STAR, mouse genome was obtained from the UCSC database (mm10) and combined with ERCC sequences while mouse gene annotation was obtained from GENCODE (VM21) and combined with ERCC annotations. ERCC sequences and annotations were downloaded from the manufacturer’s website (https://assets.thermofisher.com/TFSAssets/LSG/manuals/ERCC92.zip). Quality control of mapped RNA-seq reads was performed using Qualimap (version 2.2.1) [32]. To visualize QuantSeq signal on the UCSC genome browser, wiggle files generated by STAR were converted to bigWig file format using “wigToBigWig” obtained from UCSC after removing ERCC genome coordinates.

### PolyA site identification and gene annotation modification

GENCODE version M21 was the primary annotation used. To achieve a more comprehensive annotation of the 3’UTRs, we also incorporated the 3’UTRs annotated in RefSeq, which is obtained from the UCSC database (mm10). In addition, we identified potential polyA (pA) sites from the data. To do this, only the 3’ most nucleotide of each read is used to build a signal track for each sample. R package “derfinder” [33] was used to identify expressed regions (ERs) from these signal tracks. Specifically, an average read pile-up cutoff of 1 RPM (reads per million mapped reads) was used. ERs are annotated to gene annotations (3’UTR, 5’ UTR, exons, introns, and intergenic regions) based on GENCODE version M21, allowing for overlapping categories. ERs mapped to introns and intergenic regions are further analyzed to identify novel polyA sites. To filter for potential internal polyA priming events, sequence composition around ERs is examined. ERs with (a) matches 18-mer polyAs (with up to 6 mismatches) within 150 bp downstream from the ends; (b) matches 7-mer polyAs (with up to 1 mismatches) within 20 bp downstream from the ends; or (c) more than 50% of the polyAs within 20 bp downstream from the ends, are removed. In addition, ERs that overlap more than 20 bp with an annotated repeat region are also excluded. Filtered ERs are first mapped to RefSeq 3’UTR annotations (obtained from UCSC) and are associated with the corresponding genes. The rest of the unmapped ERs are then annotated to (a) the corresponding gene if it is intronic, or (b) the nearest gene upstream if it is within 5 kb from the gene ends. The intronic ERs are extended for 5 bp in each direction and the ERs downstream of genes are used to extend the gene’s 3’UTR annotation. The novel intronic polyA sites and extended gene annotations are then added to the gtf file used for gene counting. In total, additional internal polyA sites were added to 228 genes, and extended 3’UTRs were added to 476 genes, and 28 genes have both. Given the complexity of transcripts, the assignment to intronic polyA sites or 3’UTR extension may be impossible to distinguish in some cases. In addition, 2 of the 676 novel ERs did not make a difference to gene quantification as they overlapped with annotations from other genes.

### Gene quantification

Trimmed and filtered reads were assigned to genes using featureCounts (v1.6.2) [34] with parameters “ -s 1 -Q 255” for 3’UTR-seq.

### Processing of small RNA-sequencing reads

FastQC was used to examine the quality of sequenced reads. BBDuk (BBMap suite v37.90) was used to trim adapter sequences from reads with reference adapter sequences provided by BBMap suite and settings “hdist = 1 mink = 11” for small RNA-seq reads [35]. For miRNA size specificity, only reads less than 23 nucleotides in length were retained. Following trimming, FastQC was used to examine the quality of trimmed sequenced reads. miRDeep2 mapper.pl was used with default parameters to map reads of at least 18 nucleotides in length to the mouse genome (mm10) [36]. Known and novel miRNAs were identified using miRDeep2 main algorithm (miRDeep2.pl) with default parameters. For known miRNAs, the mature miRNA sequences in mouse were obtained from miRBase (v21) [37]. For novel miRNAs, only those with miRDeep score ≥ 2 and a sequence not matching previously reported small RNAs (rfam alert = FALSE) were retained for downstream analysis.

### mRNA and miRNA normalization and differential analysis

Low-count mRNAs and miRNAs were filtered out prior to analysis. Only mRNAs and miRNAs with normalized read count (counts per million mapped reads, CPM) > 2 in at least 10 samples were retained for downstream analysis. CPM was used because of the consistent read mapping with UTR-seq.

For mRNAs, quantification of mitochondrial genes was not considered in this study and ERCC transcripts were removed prior to differential analysis. Remove Unwanted Variation from RNA-Seq Data (RUVSeq, v1.18.0) was used with RUVg() function with empirically detected negative genes to estimate unwanted variations in mRNA data based on the previously shown superior performance of this method compared to methods using ERCC for library normalization [38]. Empirical negative-control genes were identified with an ANOVA-like test comparing all conditions (FDR < 0.1). For miRNAs, RUVSeq was used with replicates (RUVs) to normalize and remove variation between the two batches from miRNA counts [38].

Quasi-likelihood *F*-test method was used to test for differential expression of mRNAs and miRNAs with a significance cutoff of absolute fold-change (FC) > 1.5 and false discovery rate corrected (FDR) < 0.05 using edgeR (v3.26.5) [39, 40]. Both the mRNA and miRNA analyses were performed in R version 3.6.

### Novel miRNA identification

The mature sequences of novel miRNAs which were included in the miRNA differential expression analysis were used to identify homologous miRNAs in other species reported in miRBase v21 and precursor genome coordinates of the novel miRNAs were used to gain insight into their mechanism of biogenesis [41–43]. Specifically, miRNA identification with “Single sequence search” function on miRBase [44] (https://www.mirbase.org/) was used with the following parameters: “Search sequences: Mature miRNAs”, “Search method: “BLASTN”, “*E*-value cutoff: 10”, “Maximum no. of hits: 100”, “Show results only from specific organisms: Mouse”, “Word size: 4”, “Match score: + 5”, “Mismatch penalty: -4”. For miRNAs with more than one result, the miRNA with the best alignment (lowest *E*-value) is reported. If no miRNAs were identified in mouse, BLASTN was rerun with no species filter and the best alignment was reported. miRNAs with no results reported from any species are denoted with “NA”.

### Predicting transcriptional regulation using Lisa

Transcriptional regulator (TR) binding sites were predicted using Lisa [45] (http://lisa.cistrome.org/) with genes which were female- or male-biased in 2 or more ages between PD27, PD32, PD37. The full Lisa model was applied (“TR ChIP-seq Peak-RP (regulatory potential)” and “ISD-RP (in silico deletion-regulatory potential) for both motif and ChIP-seq” methods) using the DNase-seq and H3K27Ac ChIP-seq data and 3000 genes which were randomly selected as the background gene set. Results combined from H3K27ac-ChIP-seq and DNase-seq ISD models, and TR ChIP-seq peak-only models using the Cauchy combination test are shown for the ChIP-seq model.

### Pathway enrichment analysis

All pathway enrichment analyses were performed using gProfileR (v0.6.7) in R (v3.6). For differentially expressed gene pathway enrichment, all detected genes in this dataset were used as background. For miRNA–gene target pathway enrichment, all gene targets detected in this dataset were used as background with parameters “min_set_size = 3, min_isect_size = 2”.

### Co-expression module identification

Gene co-expression modules were identified using CEMitool [46] (version 1.8.2) using log2-transformed, normalized read counts with default settings. Briefly, CEMitool first uses an unsupervised method to filter for genes with sufficient variation. By default, CEMitool models the variants of the genes as an inverse gamma distribution and chooses genes with a *p* value < 0.1. Next, it automatically determines the similarity criteria before it separates genes into modules using the dynamic tree cut method. Hub genes are identified by ranking the summed similarities between a certain gene and all other genes in the same module. This analysis was performed in R version 3.6.

### miRNA–gene target correlation

Computationally predicted gene targets were curated from TargetScanMouse (v7.2) [47] and experimentally validated gene targets were curated from miRTarBase (v8.0) [48]. Only miRNA–gene target pairs from TargetScan with “Cumulative weight context score” < − 0.1 were used. “Context score” for a specific target site is defined by [47] as the summed contribution from 14 features which likely influence miRNA targeting a given gene, including “site type”, “local AU”, “3’ UTR length”, and “Probability of conserved targeting” (full feature list http://www.targetscan.org/vert_70/docs/context_score_totals.html). Spearman’s correlation coefficient (rho) was calculated for each pair using log2-transformed normalized counts and *p*-values were adjusted with FDR to account for multiple testing. Pairs were considered negatively correlated if rho < 0 and FDR-adjusted *p*-value < 0.1. This analysis was performed in R version 3.6.

### Integration of single-nuclei RNA-seq dataset from [16]

Single-nuclei RNA-seq (snRNA-seq) of 10–12 week-old snap-frozen male and female C57BL/6 mouse pituitary gland was obtained from GSE151961 and processed using Seurat 4.1.0 [49]. Data were merged from replicates for each sex separately and cells with mitochondrial gene content > 15% and ribosomal gene content > 3% were removed. The percent of mitochondrial gene content and percent of ribosomal gene content were variables regressed out of the merged data for each sex during data normalization using SCTransform [50]. Data integration between sexes was performed using Seurat with the normalized merged data [51]. Cell types were labeled by comparing gene markers identified in cell clusters calculated by Seurat using the “FindAllMarkers” functions to gene markers identified in the original paper [16]. For all downstream analyses, the “Debris” cluster, which was also identified in the original paper, was removed. This analysis was performed using R version 4.1.2. For detailed methods, our code is available at https://github.com/wilsonlabgroup/pituitary_transcriptome_analyses.

### Cell-type enrichment of co-expression module genes

A one-sided Kolmogorov–Smirnov (KS) test was performed to compare the distribution of expression of a given co-expression module gene within each cell type versus its distribution of expression values in all other cell types based on the expression data from the [16] snRNA-seq dataset (see Methods—Integration of single-nuclei RNA-seq dataset from [16] for data processing details). Resulting *p*-values were FDR-adjusted for multiple testing. Only co-expression module genes with FDR-adjusted *p*-value ≤ 0.05 in at least one cell type comparison were plotted. A one-sided hypergeometric test was then used to determine if there was enrichment for a group of module genes with statistically greater expression based on the KS test (FDR-adjusted *p*-value ≤ 0.05) in each cell type. Resulting *p-*values were FDR-adjusted for multiple testing and a group of module genes was considered to be significantly enriched if FDR-adjusted *p*-value < 0.05. This analysis was performed using R version 4.1.2.

### Proportions in admixture RNA-seq deconvolution

Proportions in admixture RNA-seq deconvolution was run as part of a wrapper script in scMappR (v1.0.7) [52] using the “compare_deconvolution_methods” function which calls the ADAPTS package (v1.0.21) [53] with RUVSeq normalized bulk counts and a custom signature matrix. In scMappR, the Proportions in Admixture method is called “WGCNA”. The custom signature matrix was generated using the “generes_to_heatmap” function from scMappR with gene markers identified from sex-integrated Ruf-Zamojski snRNA-seq data (see Integration of single-nuclei RNA-seq dataset from [16] for data processing) and selecting only the top 3000 genes with the greatest variance. This analysis was performed using R version 4.1.2.

### Identifying cell-type-specific sex-biased genes using scMappR

Cell-weighted fold-change (cwFC) of PD37 sex-biased genes was calculated using scMappR (v1.0.7) (https://cran.r-project.org/package=scMappR) with sex-integrated adult C57BL/6 mouse pituitary transcriptome [16] using the “WGCNA” (Proportions in Admixture) deconvolution method. Genes which were not detected in the single-cell reference dataset and genes which were flagged as a “false positive” by scMappR (cwFoldchange_gene_flagged_FP) were filtered out. Genes were further filtered to include outliers as determined by scMappR based on their cwFC (cwFoldchange_gene_assigned). Finally, only genes with an absolute gene-normalized cwFC > 0.5 for a given cell-type were considered cell-type-specific sex-biased genes and shown in the heatmaps. This analysis was performed using R version 4.1.2.

## Results

### Profiling postnatal mouse pituitary gland development with 3’UTR-seq and small RNA-seq

**Fig. 1:**
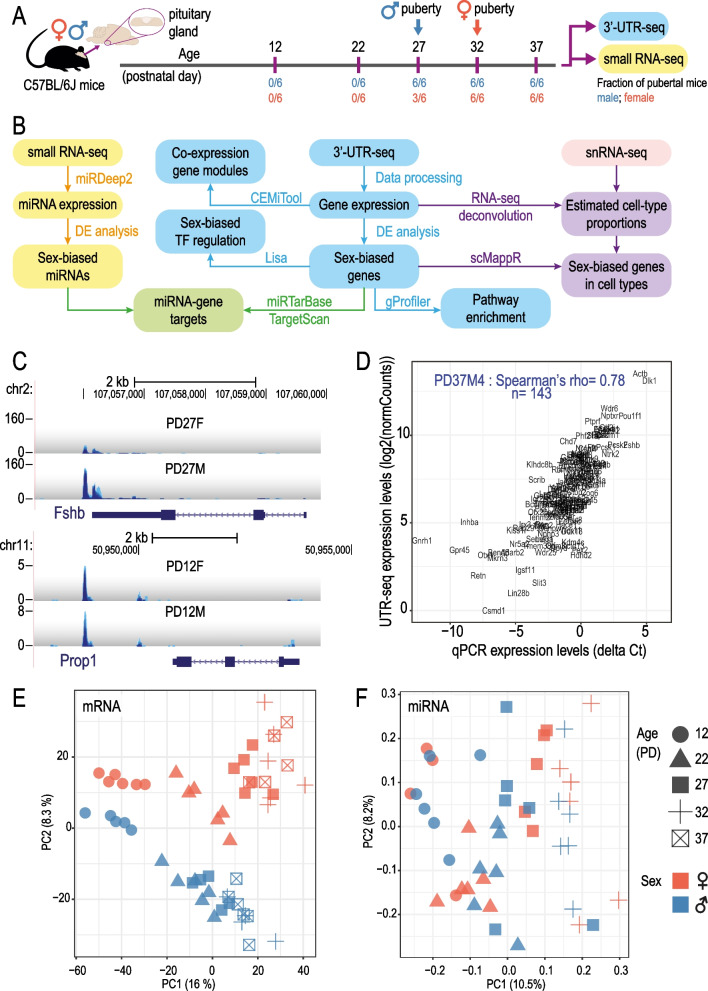
Overview of the pituitary transcriptome during postnatal development in male and female samples. **A** Schematic of experimental design. Marks (purple) on the timeline denote the age in which the pituitary gland was collected. Vertical arrows denote average ages for onset of puberty of the specified sex in our colony (determined by preputial separation for males or vaginal opening for females). Fraction of pubertal mice out of total mice for males (blue) and females (red) at each age is shown. **B** Schematic of analysis workflow. Summary of miRNA expression analyses (yellow), gene expression analyses (blue), miRNA–gene target identification (green), processing of single-nuclei RNA-seq (snRNA-seq) data from [16] (red), and combining snRNA-seq data with bulk gene expression data (purple). **C** Genome browser screenshots showing QuantSeq signal at *Fshb* and *Prop1*. *X*-axis: genomic coordinates; *y*-axis: reads per million mapped reads (RPM); *PD* postnatal day. Gene name and gene model are shown on the bottom of each panel. Each track represents overlapping signal from 5–6 biological replicates. **D** Scatter plot showing the correlation between gene quantification measured by qPCR and by 3’UTR-seq in one pituitary sample (PD37M4). *X*-axis: ΔCt values obtained by qPCR; *y*-axis: log2-transformed normalized counts (log2(normCounts)) values obtained by 3’UTR-seq. Sample name, Spearman correlation coefficient, and number of genes included are labeled on the plot. PCA plot for pituitary gland samples based on **E** gene expression and **F** miRNA expression. Principal component analysis (PCA) was performed using log2(normCounts) after filtering for low-count genes/miRNAs and normalization using RUVSeq. Only scores of the first 2 PCs are shown. Age is indicated by shape while sex is indicated by colors

To assess changes in the mouse pituitary transcriptome across postnatal development, we profiled pituitary RNA expression at 5 postnatal days spanning the pubertal transition (PD: 12, 22, 27, 32 and 37). We observed physical markers of pubertal onset, preputial separation (PS) and vaginal opening (VO) occurring on average at PD27 and PD29 in male and female mice, respectively, in our C57BL/6J colony ([54], Fig. 1A). In all analyses performed, pubertal onset refers to ages at which PS and VO were recorded (see Fig. 1B for a summary of analysis workflow).

To measure mRNA expression in a genome-wide, cost-effective, and relatively high-throughput manner, we first automated the QuantSeq 3’ mRNA-Seq protocol which profiles 3’UTR of mRNA transcripts (Methods—Library preparation and sequencing). Of the 60 libraries generated, 55 libraries were of sufficient library quantity for sequencing which resulted in 4 to 6 biological replicates for each sex at each of the five postnatal days (see Additional file 2: Table S1 for quality control metrics).

It was shown previously that single-cell-based 3’UTR profiling in the pituitary missed the expression of *Prop1* due to a lack of tissue-specific 3’UTR gene annotation [55]. To avoid similar issues with our 3’UTR profiling, we refined gene 3’-end annotations by identifying clusters of sequencing reads from our data and re-annotating 3’UTRs (Methods—PolyA site identification and gene annotation modification and Additional file 1: Fig. S1A). Improved pituitary-specific 3’UTR annotations were generated for 676 genes, allowing for assignment of significantly more reads to them, including important pituitary genes such as *Pou1f1*, *Ghrhr*, *Fshb*, and *Prop1* (Fig. 1C, Additional file 1: Fig. S1B).

To profile miRNA expression, we performed sRNA-seq in the same male and female samples used for 3’UTR-seq besides PD37 which was excluded from this experiment (PD: 12, 22, 27, and 32; *n* = 6 biological replicates; 48 libraries total). Using the miRDeep2 workflow, we identified 273 known mouse miRNAs (miRBase v21) and 19 novel miRNAs (Additional file 3: Table S2).

For both 3’UTR-seq and sRNA-seq experiments, the biological replicates were well correlated (Pearson’s correlation coefficient: 3’UTR-seq 0.95–0.97; sRNA-seq 0.86–0.90) (Additional file 1: Fig. S2A, B). Furthermore, gene expression level quantified by 3’UTR-seq correlated well with microfluidic qPCR data previously generated from the same 55 RNA samples (178 puberty-related genes plus 5 control genes) [17] (median Spearman correlation coefficient: 0.74) (Fig. 1D, Additional file 1: Fig. S1C). Using principal component analyses (PCA), we observed a separation of PD12 samples from PD22 and older for gene expression profiles along PC1 (Fig. 1E). Although more subtle, we observed samples distributed by age along PC1 for miRNA expression profiles (Fig. 1F). We also observed separation between male and female samples along PC2 at all ages based on gene expression profiles, which became more pronounced across postnatal ages (Fig. 1E). In contrast, no obvious sex differences were observed in our miRNA expression data (Fig. 1F).

### Sex-biases in the transcriptome occur prior to puberty

**Fig. 2:**
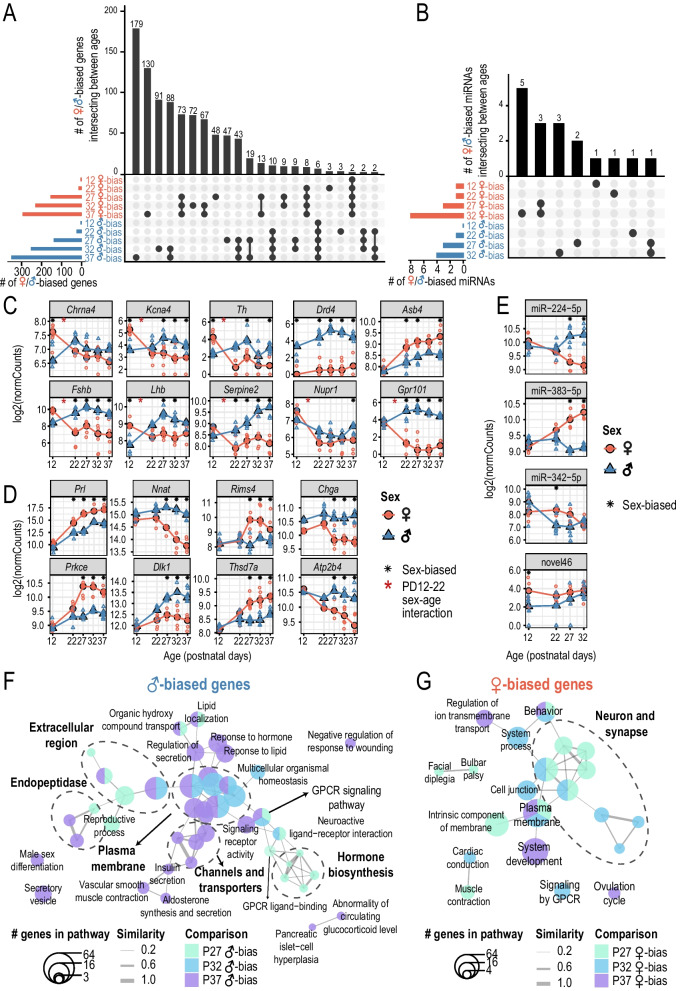
Pituitary transcriptome is increasingly sex-biased across postnatal development. Barplot showing the number of **A** intersecting sex-biased genes or **B** intersecting sex-biased miRNAs between each age. Horizontal bars on the bottom left side of each plot show the numbers of male- (blue) or female-biased (red) genes/miRNAs at each age (absolute FC > 1.5; FDR < 0.05). Different intersection combinations between sex-biased genes/miRNAs identified at each age are represented by the dotplot. The number of genes/miRNAs which intersect in the indicated combination of sex comparisons is shown by the vertical barplots (# overlapping sex-biased genes/miRNAs). Expression plots of example **C** pre-pubertal (PD12-22) sex-biased genes and genes with sex-by-age effect between PD12 and PD22, **D** peri-/post-pubertal sex-biased genes, and **E** sex-biased miRNAs. Log2-transformed normalized counts (log2(normCounts)) are plotted for each gene/miRNA. Expression changes are shown across ages (x-axis). Large, filled points represent median expression at each age and unfilled points represent each biological replicate. Blue: male samples; red: female samples. Black asterisks highlight ages at which the corresponding genes/miRNAs are detected as sex-biased and red asterisks highlight genes with significant sex-by-age effect between PD12 and PD22. Network representation of pathways enriched for **F** male-biased and **G** female-biased differentially expressed genes. Each node represents a pathway and nodes are connected based on similarity in genes found enriching for the connected pathways (Jaccard distance). Node size represents the number of differentially expressed genes enriching for the given pathway and nodes are colored based on the differential expression comparison in which the genes were identified. Pathways that share similar genes are circled (dashed lines) and labeled manually based on pathway functions. Specific pathways and their manual labels can be found in Additional file 6: Table S5

We quantified sex differences in the pituitary transcriptome by comparing the expression of genes and miRNAs between male and female samples at each age. Across all the profiled ages, we observed an increase in the numbers of sex-biased genes and miRNAs, with the most dramatic increase occurring at PD27, when all the males and half of the females had gone through puberty as measured by PS and VO (Figs. 1A, 2A, B see Tables 1, 2 for list of significant sex-biased genes and miRNAs; see Additional file 4: Table S3, Additional file 5: Table S4 for full list of differential analysis results).

**Table 1:** Sex-biased genes at each profiled age

	PD12	PD22	PD27	PD32	PD37
Female-biased	*Xist, Lhb, Kdm6a, Kcna4, Chrna4, Th*	*Xist, Prl, Kdm6a, Greb1, Asb4, Cdkl4, Ecel1, Smyd3, Gm29374, Dok7, Gucy2f, Kcnd3, Gm47163, Minar1, Trim9, Cdkn1c*	*Xist, Prl, Prkce, Gm29374, Cdkl4, Greb1, Ecel1, Pcdh10, Ntm, Nav2, Nrxn3, Znrf2, Gm47163, F730016J06Rik, Gucy2f, Gad2, Pcdh11x, Csrnp3, Grm5, Phldb2, Asb4, Ankrd34a, Hmcn1, Scn11a, Shc2, Rtn1, Akr1c14, Ar, Thsd7a, Gm32849, Gm15408, Pak7, Rxfp1, Chml, Nhlh2, Kctd4, Pianp, 4933406B17Rik, Ccdc116, Ank1, Gm15411, AC121821.1, Dlgap1, Calb1, Zfp804a, Pvalb, Fbxo31, Opn3, Dmrta1, Sh3d19*	*Xist, Akr1c14, Lpl, Gm47163, Prl, Nrxn3, Pcdh11x, Ntm, Phldb2, Ar, Greb1, Rabgap1l, Reln, Prkce, Pcdh10, Cdkl4, Pde7b, Pak7, Chml, 4933406B17Rik, Rxfp1, Crhbp, Nrxn2, Dennd2a, Chst8, Tspan7, Ecel1, Gm21846, Grm5, Neurod4, Pcdh9, Cadps2, Apbb1, Thsd7a, Klhdc8b, Dmrta1, Hmcn1, Opn3, Crtac1, Znrf2, Csrnp3, Nhlh2, Gucy2f, Rasa4, Tiam1, Wdr89, Plppr3, Cryz, Slc35f1, Pgr*	*Xist, Pcdh11x, Prl, Gucy2f, Dmrta1, Cdkl4, Dclk1, Akr1c14, Pak7, Chml, Gm29374, Ntm, Ar, Greb1, Rabgap1l, Id2, Gm21846, Slc35f1, Reln, Grm5, Id1, Kdm6a, Tent5a, Cadps2, Asb4, Prkce, Gm47163, Nhlh2, Hmcn1, 4933406B17Rik, Pcdh10, Flrt1, Phldb2, Sh3d19, Nrip1, Six6, Neurod4, Opn3, Ecel1, Rxfp1, Crhbp, 5031439G07Rik, Rtn2, Zfp804a, Il6st, Igf1r, Ank1, Tmem266, Ddx3x, Lrp1b*
Male-biased	*Ddx3y, Uty, Gm29650, Kdm5d, Eif2s3y, Drd4*	*Ddx3y, Uty, Gm29650, Kdm5d, Eif2s3y, Dgkk, Gpr101, Drd4, Nmu, Pcsk1, Ptx4, Fshb, Nkain1, Ly6h, Tspan12, Fam167b, Osbp2, Lcn2, Acadl, Cyb5r2, Myo1d, Serpine2, Gpd1, AC117700.1, Tspan4*	*Ddx3y, Uty, Gm29650, Kdm5d, Eif2s3y, Drd4, Dgkk, Gpr101, Nnat, Pcsk1, Ramp3, Dlk1, Lhb, Ret, Scd1, Timp1, Dapl1, Homer2, Cecr2, Rbp4, Ostf1, Card19, Chga, Mmp20, Liph, Fetub, Rab3b, Ptx4, Atp2b4, Kcnmb4, Smox, Serpina3c, Serpine2, Cidea, Spp1, AC117700.1, Cd109, Gabre, Nmu, Cga, Pappa2, Kcnmb4os2, Nckap1l, Arhgap36, H2-Eb2, Th, Gm13889, Cpm, Jam3, Ell2*	*Ddx3y, Uty, Gm29650, Kdm5d, Eif2s3y, Dapl1, Nnat, Gpr101, Dlk1, Drd4, Scd1, P4ha2, Arhgap36, Homer2, Serpine2, Pappa2, Rab3b, Tacc2, Galnt16, Rbbp8, Fads1, Nrip3, Ret, Fetub, Slc39a14, Mgat4c, Serpina3c, Nmnat2, Fkbp5, Lhb, Arhgef3, Ramp3, Vangl1, Kcna5, Ptx4, Dapp1, Ppl, Gabre, Cidea, Atp2b4, Smim1, Gh#, Dgkk, Aldh1b1, Tspan12, Tmem52, Car10, Tmc3, Sytl3, Chga*	*Ddx3y, Uty, Gm29650, Kdm5d, Eif2s3y, Homer2, Dlk1, Dapl1, Serpine2, Nnat, Arhgap36, Gpr101, Drd4, Atp2b4, Dapp1, Grb10, Kcnk9, Sytl4, Fetub, Pappa2, P4ha2, Mrap2, Scd1, Aldh1b1, Igsf1, Jam3, Tacc2, Ntrk3, Lgi3, Tgm2, Deup1, Galnt10, Ramp3, Scg2, Nmnat2, Olfml2b, Chga, Pcsk2os1, Smim1, B4galt1, Ddc, Nckap1l, Fam20c, Pcsk1, Entpd3, Grip2, Lpin1, Fyb2, BC039966, Crispld2*

Genes are ranked by false discovery rate followed by fold-change (FDR < 0.05, absolute FC > 1.5) (capped at 50 genes per comparison)

**Table 2:** Sex-biased miRNAs at each profiled age

	PD12	PD22	PD27	PD32
Female-biased	novel46	miR-342-5p	miR-383-5p, miR-30d-5p, miR-17-5p	miR-383-5p, miR-205-5p, miR-146a-5p, miR-17-5p, miR-485-5p, miR-30d-5p, novel184, miR-669f-5p
Male-biased	NA	miR-499-5p	miR-224-5p, miR-181a-5p, miR-365-2-5p	miR-224-5p, novel37, miR-871-3p, miR-30b-5p

miRNAs are ranked by false discovery rate followed by fold-change (FDR < 0.05, absolute FC > 1.5). No results are shown for PD37 as this age was not profiled by sRNA-seq. NA indicate no sex-biased miRNA was identified

Although we observed most sex-biased gene expression at peri- and post-pubertal ages, sex differences in the pituitary begin to manifest earlier in postnatal development at PD12 (Table 1). At PD12, 12 genes, including seven sex chromosome-linked genes: *Ddx3y*, *Uty*, *Kdm5d*, *Gm29650*, *Eif2s3y* (Y-linked), *Xist* and *Kdm6a* (X-linked); and five autosomal genes, *Chrna4*, *Kcna4*, *Lhb*, *Th*, and *Drd4*, are identified as significantly sex-biased (FDR < 0.05, absolute fold-change > 1.5) (Fig. 2C, Additional file 1: Fig. S3A). The seven sex chromosome-linked genes were male- or female-biased across all five profiled ages (PD12 to PD37). Other than *Kdm5d*, *Ddx3y*, *Eif2s3y*, *Uty*, *Xist*, and *Lhb* [9, 18, 56], sex differences in the expression of other sex-biased genes we detected at PD12 have not been reported previously in pituitary.

We identified 25 male-biased and 16 female-biased genes at PD22 (Table 1), an age preceding puberty, including puberty-related genes *Dgkk*, *Fshb*, *Osbp2*, and *Pcsk1*, which we previously identified as sex-biased using microfluidic qPCR [17] (Fig. 2C, Additional file 1: Fig. S3A). Of these 41 sex-biased genes at PD22, we found 18 genes which start to exhibit sex-biased expression at PD22 and maintain the same sex-biased trend at all older ages we profiled, (e.g., *Asb4* and *Serpine2*) (Fig. 2A, C, Additional file 1: Fig. S3A), demonstrating that there is establishment of sex-biased expression in the pituitary prior to puberty.

We noticed some genes exhibited sex-biased changes between PD12 and PD22 (e.g., *Fshb*). We then specifically tested for sex-by-age interaction effect, and identified 13 genes (*Fshb*, *Pcsk1*, *Serpine2*, *Lhb*, *Chrna4*, *Nupr1*, *Dgkk*, *Steap3*, *Timp1*, *Kcna4*, *Gpr101*, *2010007H06Rik*, and *Th*) with significant sex-by-age interaction effect between PD12 and PD22 (FDR < 0.05). All of these genes showed similar or increased expression in females compared to males at PD12 but displayed decreased expression in females compared to males at PD22 (Fig. 2C, Additional file 1: Fig. S3A).

For miRNAs, we identified 3 miRNAs with sex-biased expression prior to puberty (PD12 or PD22). The one sex-biased miRNA at PD12 displayed female-biased expression and was one of 20 miRNAs (after normalization and filtering out low-count miRNAs; Additional file 3: Table S2) not previously identified in mice based on miRBase v21 (we tentatively named it novel46; Fig. 2E, Table 2, Additional file 1: Fig. S3B). We did not observe any alignments of novel46 to miRNAs in other species found in miRBase v21. Based on its miRNA precursor coordinates, novel46 is a mirtron (a miRNA which is spliced from a host gene intron) expressed from the last intron of calcium voltage-gated channel subunit alpha1 G (*Cacna1g*), located on chromosome 11 (Additional file 3: Table S2, Additional file 1: Fig. S3C).

At PD22, we found that miR-499-5p displayed male-biased expression, and based on its miRNA precursor coordinates, miR-449-5p is a mirtron expressed from *Myh7b* (Table 2, Additional file 1: Fig. S3B). We also found that miR-342-5p displayed female-biased expression at PD22, and based on its miRNA precursor coordinates, miR-342-5p is a mirtron expressed from *Evl* (Fig. 2E, Table 2, Additional file 1: Fig. S3B). However, neither *Myh7b* nor *Evl* were detected as significantly sex-biased at any profiled age.

### Peri- and post-pubertal sex differences in gene expression reflect sex differences in pituitary endocrine functions

At peri- and post-pubertal stages, we detected similar numbers of male- and female-biased genes. The number of sex-biased genes roughly doubled across the pubertal transition (140, 253, and 351 male-biased genes and 156, 232, and 294 female-biased genes at PD27, PD32, and PD37, respectively; Fig. 2A, Table 1, Additional file 4: Table S3). Many of these genes (43 male and 73 female-biased) showed sex-biased gene expression throughout the pubertal transition (across PD27, PD32, and PD37) (Fig. 2A, D, Additional file 1: Fig. S3A). While we recovered genes with previously known sex-biased expression in pituitary, such as *Dlk1* and *Prl* [9, 17, 57], many other genes whose sex-biased expression has not been previously identified in the pituitary were found (Fig. 2D, Table 1), including the male-biased expression of an abundant transcript in the pituitary, neuronatin (*Nnat*) [58], at PD27, PD32 and PD37.

Male-biased genes are enriched for pathways related to hormone synthesis and secretion (Fig. 2F, Additional file 6: Table S5), including “peptide hormone biosynthesis” (PD27, *p* = 7.22e−06), “regulation of secretion” (PD37, *p* = 7.87e−03), and “secretory vesicle” (PD37, *p* = 4.9e−04); and pathways related to reproduction, including “male sex differentiation” (PD37, *p* = 4.76e−02) and “reproductive process” (PD27, *p* = 4.14e−02). In addition, male-biased genes are enriched for pathways associated with signaling receptors, ion channels, extracellular region, and plasma membrane, like “G-protein coupled receptor signaling pathway”, which is enriched at all three ages (PD27: *p* = 4.7e−02; PD32: *p* = 1.22e−03; PD37: *p* = 8.67e−03). Finally, pathways related to endopeptidase inhibitor activity are also enriched, including several serpin family genes (*Serpine2*, *Serpina3c*, and *Serpinb1a*).

Female-biased genes, including *Stat5a*, *Cckbr*, *Slit2*, *Robo2*, *Nrip1*, *Nhlh2*, *Prl*, and *Pgr*, are enriched for “*ovulation cycle*” (PD37: *p* = 1.03e−02), linking female-biased pituitary genes to female-specific physiological processes. Notably, other female-biased pathways are predominantly neuron-related (Fig. 2G, Additional file 6: Table S5); this could be attributed to genes expressed in the posterior pituitary, which contains axons extended from the hypothalamus, or genes expressed in neuroendocrine cells, which are known to be activated in a neuron-like manner [59–61]. Particularly, female-biased genes at all three ages, including *Crhbp*, *Prl*, *Calb1*, *Pgr*, *Pak7*, *Reln*, *Dmrta1*, *Prkce*, *Ar*, *Nhlh2*, *Grm5*, and *Cacna1c*, are enriched for “*behavior*” (PD27: *p* = 5.32e−03; PD32: *p* = 4.25e−02; PD37: *p* = 5.32e−03). Several of these genes, *Crhbp* [62], *Prl* [63], *Calb1* [64], *Reln* [65], *Prkce* [66], *Ar* [67], *Nhlh2* [68], *Grm5* [69], and *Cacna1c* [70], are related to the regulation of stress, which is sex-biased in its activity [71].

For miRNAs, we detected 3 and 4 male-biased miRNAs and 3 and 8 female-biased miRNAs at PD27 and PD32, respectively (Fig. 2B, Table 2, Additional file 1: Fig. S3B, Additional file 5: Table S4). miR-224-5p, a mirtron of *Gabre*, and miR-383-5p, a mirtron of *Sgcz*, respectively, displayed male- and female-biased expression levels at both PD27 and PD32 (Fig. 2E, Table 2).

### Connecting sex-biased miRNAs to target genes with sex-biased expression

**Fig. 3:**
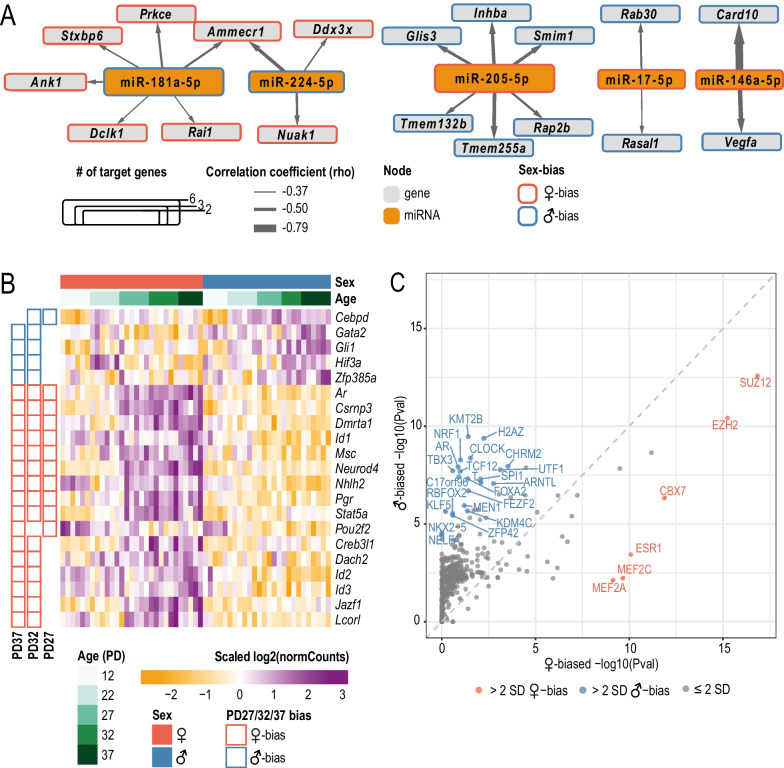
Regulation of sex-biased pituitary gene expression by miRNAs and transcriptional regulators (TRs). **A** Interaction network of sex-biased miRNAs and their sex-biased target genes for all ages. Each node represents a gene (gray) or a miRNA (orange) with the node size representing its number of connections with other nodes. Outlines on nodes indicate gene/miRNA is female-biased (red) or male-biased (blue). Only miRNAs with 2 or more connections are shown. Edges between each node show a predicted interaction between the miRNA and gene. Edge thickness indicates Spearman’s correlation coefficient (rho) calculated for the given pair. **B** Heatmap of sex-biased pituitary TR gene expression. TRs which are sex-biased in the same direction at a minimum of two ages between PD27, PD32, and PD37 are plotted. Colors of the heatmap represent row-scaled and centered expression levels of each gene. Column annotation bars indicate sample age and sex. Row annotation bars indicate age at which the gene was found to be sex-biased. **C** Scatterplot comparing Lisa TR rankings with combined P-values predicted to regulate female- and male-biased genes. Each TR is represented by a point. The combined − log10(*P*-value) is plotted for each TR based on female-biased and male-biased gene sets which are sex-biased in the same direction at a minimum of two ages between PD27, PD32 and PD37. Colored points show TRs which have change in − log10(*p*-value) between sexes which is two standard deviations greater than the mean change in − log10(*p*-value). Red points indicate TRs which are enriched for regulating female-biased genes; blue points indicate TRs which are enriched for regulating male-biased genes

To evaluate post-transcriptional regulation of sex-biased changes in the pituitary gland by miRNAs, we identified computationally predicted or experimentally validated miRNA–target gene pairs that both exhibit sex-biased expression. Since miRNAs are usually predicted to repress target gene expression, we additionally required miRNA and target gene pairs to be significantly negatively correlated in expression across matched samples (Spearman’s rho < 0, FDR < 0.1) (see Methods—miRNA–gene target correlation for details) (Fig. 3A, Additional file 7: Table S6).

At pre-pubertal ages, PD12 and PD22, we did not identify any negatively correlated miRNA–gene pairs where the miRNA and its target gene were sex-biased. This is likely due to the relatively lower number of sex-biased genes and miRNAs identified at these earlier profiled ages.

At peri- and post-pubertal ages, we found 18 putative sex-biased gene targets of 5 sex-biased miRNAs. For example, the male-biased miRNAs, miR-181a-5p (at PD27) and miR-224-5p (at PD27 and PD32), showed significant negative correlation with female-biased target genes, *Ammecr1* (target of miR-181a-5p and miR-224-5p), *Ank1* (target of miR-181a-5p), and *Prkce* (target of miR-181a-5p) (Fig. 3A, Additional file 7: Table S6). In comparison, female-biased miR-205-5p (at PD32) was negatively correlated with *Inhba* (Fig. 3A, Additional file 7: Table S6).

### Predicting transcriptional regulators associated with sex-biased pituitary gene expression

Sex-biased expression of transcriptional regulators (TRs) is a principal mechanism by which sex-biased regulatory networks can be generated. Of the sex-biased genes we detected, 5 male-biased and 16 female-biased genes are annotated as TRs in mouse by AnimalTFDB3 [72] (Fig. 3B). Known molecular functions in the pituitary and pituitary-related knockout phenotypes of these 21 sex-biased TRs are summarized in Table 3.

**Table 3:** Sex-biased TRs and their pituitary-related phenotypes and functions

	Gene name	Gene description	Known pituitary functions	Mutant pituitary phenotype	References
Male-biased	*Cebpd*	CCAAT/enhancer binding protein (C/EBP), delta	Suppresses prolactin expression	NA	[98]
	*Gata2*	GATA binding protein 2	Specification/expansion of thyrotropes; maintenance of hormone production in gonadotropes and thyrotropes	Pituitary specific knockout: decreased thyrotropes population at birth; transient developmental delay in males; lower level of FSH and TSH in adults	[93]
	*Gli1*	GLI-Kruppel family member GLI1	Involved in cell proliferation, hormone release, CRH signaling transduction in adult pituitary	NA	[99, 100]
	*Hif3a*	Hypoxia inducible factor 3, alpha subunit	Downregulated in gonadotrope nonfunctioning pituitary adenomas	NA	[101]
	*Zfp385a*	Zinc finger protein 385A	NA	NA	
Female-biased	*Ar*	Androgen receptor	Regulates LH and FSH expression and secretion; maintains the negative feedback system of glucocorticoid production	Pituitary specific knockout: lower FSH serum levels and reduced LH surge in female mice; full knockout: increased proopiomelanocortin (POMC) and decreased glucocorticoid receptor (GR) expression	[79, 102]
	*Creb3l1*	cAMP responsive element binding protein 3-like 1	Regulates the expression of *Pcsk1* in corticotroph cell line AtT20; Regulates the expression of transport factors and induces Golgi complex expansion in response to stimuli in secretory cells	NA	[103, 104]
	*Csrnp3*	Cysteine-serine-rich nuclear protein 3	NA	NA	
	*Dach2*	Dachshund family transcription factor 2	NA	NA	
	*Dmrta1*	doublesex and mab-3 related transcription factor like family A1	NA	NA	
	*Id1-3*	Inhibitor of DNA binding 1–3	Induced and likely modulates gene expression in melanotropes under constant stress	NA	[105]
	*Jazf1*	JAZF zinc finger 1	NA	NA	
	*Lcorl*	ligand dependent nuclear receptor corepressor-like	NA	NA	
	*Msc*	Musculin	NA	NA	
	*Neurod4*	Neurogenic differentiation 4	Required for somatotrope differentiation	Full knockout: decreased somatotropes, minimal expression of GHRHR	[106]
	*Nhlh2*	Nescient helix loop helix 2	Plays a role in regulation of gonadotropins and GnRH receptor expression	Full knockout: impaired pubertal development in female mice	[107]
	*Pgr*	Progesterone receptor	Likely plays a role in regulating LH surge	NA	[108, 109]
	*Pou2f2*	POU domain, class 2, transcription factor 2	NA	NA	
	*Stat5a*	Signal transducer and activator of transcription 5A	NA	NA	

TRs included displayed sex-biased gene expression (FDR < 0.05, absolute FC > 1.5) in at least two post-pubertal ages (PD27, PD32 or PD37). NA indicates that currently there is no known pituitary function or mutant pituitary phenotype

To gain insights into how TR proteins might regulate sex-biased pituitary gene expression across pubertal transition, we used the bioinformatic tool Lisa (epigenetic Landscape In Silico deletion Analysis; [45]). Lisa takes a list of genes and builds transcriptional regulatory models based on both publicly available DNase and ChIP-seq datasets and returns predictions of candidate TRs that regulate them. To focus on the major increase in sex-biased gene expression occurring at peri-pubertal and post-pubertal ages (PD27, PD32, and PD37), we provided Lisa with pituitary genes classified as having sex-biased gene expression at two or more postnatal days between PD27, PD32, and PD37 in males (*n* = 183) or females (*n* = 174). We identified 6 and 22 TRs (Δ-log10(*p*-value) > mean ± 2SD) including gonadal nuclear hormone receptors, ESR1 and AR, that were predicted by Lisa to regulate female-biased and male-biased genes, respectively. In addition, CBX7, a component of Polycomb repressive complex 1 (PRC1), as well as SUZ12 and EZH2, components of Polycomb repressive complex 2 (PRC2), were predicted by Lisa to regulate female-biased genes. In contrast, a heterodimeric TR, CLOCK:BMAL1 (ARNTL), was predicted by Lisa to regulate male-biased gene expression (Fig. 3C).

### Gene co-expression analysis reveals dynamic modules enriching for pituitary cell types

**Fig. 4:**
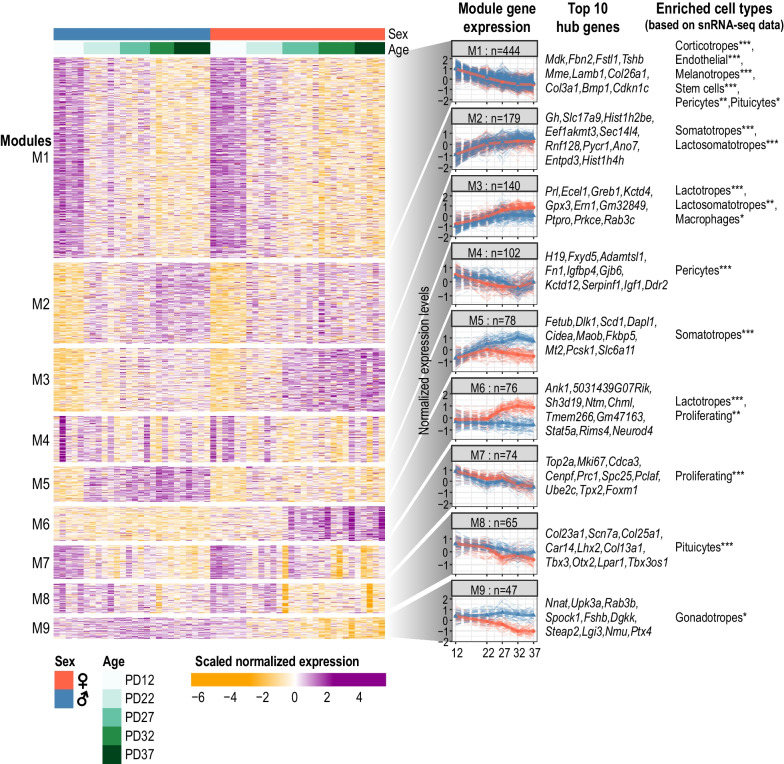
Co-expression network analysis identifies gene modules underlying pituitary transcriptome changes. Heatmap shows genes selected for co-expression analysis, separated into 9 modules. Column annotation bars indicate sample age and sex. Colors of the heatmap represent row-scaled and centered expression levels of each gene. A summary of the expression profiles of each module is shown in the left column of the right panel. The solid line represents the median expression (scaled and centered as shown in the heatmap) at each age and sex (red: female samples, blue: male samples) for all the genes in the corresponding module. Dash lines represent the scaled expression profiles of each gene in the module. Module names and number of genes included in the module are labeled. Top 10 hub genes (calculated based on genes’ connectivity within the modules) are listed for each module. All co-expressing module genes are listed in Additional file 8: Table S7. Cell types in which module genes are enriched in based on single-nuclei RNA-seq expression from [16] are shown on the right (see Additional file 1: Fig. S6C and Methods—Cell-type enrichment of co-expression module genes for details). One-sided hypergeometric test: * FDR < 0.05, ** FDR < 0.01, *** FDR < 0.001

To further characterize developmental changes in the pituitary transcriptome, we utilized our temporal and sex-dependent gene expression profiling of the postnatal pituitary to build gene co-expression networks because co-expressing genes tend to share related functions or regulatory pathways. We used CEMiTool [46] to identify transcriptome-wide gene expression correlation networks in the postnatal pituitary transcriptome (Methods—Co-expression module identification). In total, nine co-expression gene modules were identified based on the expression of 1205 genes (Fig. 4, Additional file 1: Figs. S4, S5, Additional file 8: Table S7).

The temporal expression profiles of the 9 co-expression gene modules showed three general patterns: (1) decreasing expression, particularly between PD12 and PD22 (Modules 1 (M1), M4, M7, and M8); (2) increasing expression (M2 and M3); and (3) sex-biased expression (M5, M6, and M9) (Fig. 4, Additional file 1: Fig. S5A-B).

To further understand the nature of the co-expression modules, we used three independent approaches: (1) pathway enrichment analysis; (2) hub gene identification; and (3) an enrichment test where we ask if a set of genes in a module show enhanced expression in specific adult pituitary cell types (previously determined by single-nuclei RNA-seq (snRNA-seq) [16]).

Using this approach, we found that our co-expression modules contain clear cell-type-enriched gene expression signatures. For example: (1) the M7 module, shows decreased expression during postnatal development in both males and females and is enriched for cell cycle-related pathways (Additional file 1: Fig. S5C); (2) the top M7 module hub genes include *Top2a*, *Mki67*, *Cdca3*, *Cenpf*, and *Prc1*, all of which are canonical markers of proliferating cells (Additional file 1: Fig. S4); and (3) using published snRNA-seq datasets of adult female and male mouse pituitary gland [16] (Additional file 1: Fig. S6A-B), we found that there is a significant enrichment of M7 genes in the proliferating cell population (Hypergeometric test, FDR = 2.60e-41) (Fig. 4, Additional file 1: Fig. S6C, Methods—Cell-type enrichment of co-expression module genes).

Similarly, the top hub genes for M2 and M5 include *Gh* and *Dlk1* (Fig. 4), markers of somatotropes [57], and there was a significant enrichment of both M2 (FDR = 6.84e−11) and M5 (FDR = 2.82e−10) module genes in somatotropes (Additional file 1: Fig. S6C). The top hub gene of M3 is *Prl*, a marker of lactotropes, and there is a significant enrichment of M3 module genes (FDR = 3.18e−24) in the lactotropes. Finally, the top hub genes in M8 consist of pituicyte markers, including *Col13a1*, *Scn7*, and *Col25a1* [73], there is also a significant enrichment of M8 module genes (FDR = 1.61e−17) within the pituicytes (Additional file 1: Fig. S6C). Overall, we observe a clear association between cell types and specific gene co-expression modules identified in the pituitary gland.

### Sex differences in cell proportions emerge prior to and across puberty

**Fig. 5:**
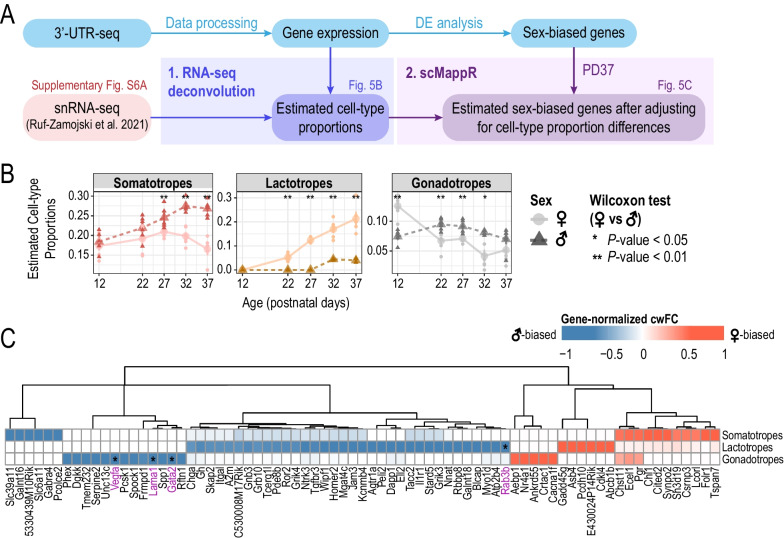
Estimating sex differences in pituitary cell types by leveraging single-nuclei RNA-seq. **A** Schematic of analysis workflow for estimating sex differences in pituitary cell types using bulk gene expression with single-nuclei RNA-seq data from [16]. **B** Estimated cell-type proportions by RNA-seq deconvolution using Proportions in Admixture (WGCNA) changes across profiled ages of cell types with previously established sex-biased proportions in the adult pituitary. Estimated cell-type proportions are plotted across postnatal ages along the *x*-axis. Large circles and triangles represent the mean cell-type proportion at each age and small circles and triangles represent each biological replicate. Lighter color, solid line, circle points: female samples; dark color, dotted line, triangle points: male samples. Wilcoxon test was performed to compare cell proportions between both sexes at each age (*p < 0.05, **p < 0.01). See Additional file 1: Fig. S6D for all other pituitary cell type proportions. **C** Heatmap of cell-weighted fold-changes (cwFC) for sex-biased genes at PD37 in somatotropes, lactotropes, and gonadotropes. Color gradient indicates gene-normalized cwFC value calculated by scMappR; red: more female-biased; blue: more male-biased. Genes with |gene-normalized cwFC|> 0.5 in at least one cell type are plotted. Genes shown to be sex-biased in the same direction in the same cell-type by [14] are indicated by the purple text and an asterisk within the cell of the corresponding cell-type

Although sex differences in the number of somatotropes, lactotropes and gonadotropes have been documented in the adult pituitary gland [15, 16, 74], much less is known about when these sex differences arise during postnatal development. To assess whether sex differences in cell proportions of somatotropes, lactotropes and gonadotropes are dynamic across pubertal development, we estimated cell-type proportions in our temporal bulk RNA-seq using the Proportions in Admixture RNA-seq deconvolution algorithm [75] from Automated Deconvolution Augmentation of Profiles for Tissue Specific cells (ADAPTS) [53] (Fig. 5A, B, Additional file 1: Fig. S6D). All cell types detected in the sex-integrated single-nuclei adult female and male pituitary transcriptome were used as our reference [16] (Additional file 1: Fig. S6A, B).

We found that we were able to recapitulate known sex biases in adult pituitary cell-type proportions [15, 16, 74] at our oldest profiled age, PD37, where the estimated cell-type proportions for somatotropes were significantly male-biased, and lactotropes were significantly female-biased (Fig. 5B). In addition, we identified sex differences in estimated cell-type proportions which were dynamic across our earlier profiled ages. For example, we observed significant sex differences in estimated gonadotrope cell-type proportions between PD12 and PD22, ages prior to puberty, and these proportions show a sex-by-age trend (from PD12 to PD22, decreasing proportions in females and increasing proportions in males), which could contribute to some of the sex-by-age bulk gene expression we observed (Fig. 5B). At PD27, when puberty has occurred in a subset of our mice (Fig. 1A), we observed the emergence of male-bias in somatotrope cell proportions and female-bias in lactotrope cell proportions (Fig. 5B).

### Inferring sex-biased gene expression in pituitary cell types

We next examined if any sex-biased genes identified remained sex-biased after adjusting for cell-type proportion sex differences in somatotropes, lactotropes, and gonadotropes. To do this, we used a bioinformatic pipeline, Single-cell mapper (scMappR) [52] to calculate a cell-weighted fold-change (cwFC) for each sex-biased gene identified in our bulk RNA-seq data (Fig. 5A). Briefly, cwFCs were calculated for each sex-biased gene in somatotropes, lactotropes, and gonadotropes, by readjusting the bulk gene expression fold-change with the cell-type specificity of the gene and the ratio of deconvolution-estimated cell-type proportions (determined by snRNA-seq). To minimize potential confounding effects from differences in ages, we focused on sex-biased genes identified at PD37, which is the closest age to the snRNA-seq reference dataset from adult male and female mice [16]. We identified 75 sex-biased genes which remain sex-biased after adjusting for sex differences in cell-type proportions (|cwFC|> 0.5) across somatotropes, lactotropes, and gonadotropes (Fig. 5C, Additional file 9: Table S8). Of these 75 sex-biased genes, 4 genes (*Gata2*, *Rab3b*, *Vegfa*, and *Lama1*) were found to be concordantly sex-biased in the corresponding cell type in the rat anterior pituitary (genes highlighted in Fig. 5C, [14]). Thus, by combining our temporal postnatal bulk pituitary 3’UTR-seq with a snRNA-seq dataset we can infer postnatal pituitary cell-type specificity of sex-biased genes and gain insights into sex-biased temporal gene regulation in a cell-type-specific manner.

## Discussion

The pituitary gland displays sex differences in its regulated physiological functions, including stress response, somatic growth, reproduction, and pubertal timing. In our previous work, we showed by qPCR profiling that selected genes associated with the onset of puberty were increasingly sex-biased with pubertal development in the mouse pituitary gland [17]. In this study, we aimed to further understand if sex bias exists beyond puberty-related genes and identify potential regulatory mechanisms. By comparing transcriptome-wide pituitary gland gene and miRNA expression profiles between male and female mice during postnatal development, we have identified sex-biased genes and miRNAs that contribute to sex differences in postnatal pituitary development and function.

While the majority of sex differences in pituitary gene expression were observed at puberty and beyond, we observed robust sex-biased expression of genes and miRNAs prior to onset of physical markers of puberty (VO/PS) at PD12 and PD22. These sex differences may be attributed to irreversible organizational effects [76] that are established by gonadal hormone exposures during fetal development and perhaps in the first week after birth in mice [64]. Unlike activational effects, sex differences established by organizational effects persist even when gonadal hormones are present at low levels in the system, such as at pre-pubertal ages. We found that many sex-biased genes identified at PD12 or PD22 or genes showing sex-by-age effect between PD12 and PD22 are expressed in the gonadotropes, including *Fshb* and *Lhb* (which encode for β-subunits of gonadotrope-secreted hormones), *Chrna4* (which is a marker for gonadotropes in rats [14]), *Nupr1* (which is involved in embryonic gonadotrope development [77]), as well as *Gpr101* and *Serpine2* (which display sex-biased gene expression in gonadotropes isolated from juvenile mice but not adult mice [18]). Our RNA-seq deconvolution analyses also suggest that gonadotrope cell proportions are male-biased prior to puberty. As gonadotropes produce LH and FSH to regulate gonadal maturation during puberty, these pre-pubertal sex differences in gonadotrope cell proportions and sex-biased expression suggest that these sex differences may be necessary to prime the pituitary gland for its regulation of gonadal maturation during puberty.

By identifying a list of sex-biased genes, we were able to predict trans regulators that are likely to underlie their expression. Expectedly, we predicted by Lisa that ESR1 and AR are involved in regulating female- and male-biased gene expression. However, since Lisa does not differentiate between activating and repressing effects of TRs on gene expression, Lisa-predicted regulation of male-biased genes by AR suggests that AR is either activating their expression in males or is repressing their expression in females. Our observation of female-biased *Ar* gene expression suggests the latter. Pituitary-specific loss of AR in male mice result in increased *Prl* gene expression and serum PRL levels [78], and gonadotrope-specific loss of AR in female mice result in decreased *Fshb* gene expression and serum FSH and LH levels [79]. In addition, global AR ablation affects its repression of sex-biased genes in the liver by altering methylation levels at the promoter regions [80], whether this has a similar effect on pituitary sex-biased gene expression remains to be examined. We also predicted by Lisa that female-biased gene expression involved regulation by polycomb complexes PRC1 (CBX7) and PRC2 (SUZ12 and EZH2). This observation is consistent with results obtained from the GTEx consortium who compared adult human male and female pituitary gene expression [13]. Mechanistically, sex-biased deposition of H3K27me3 marks by PRC2 through its catalytic subunits Ezh1/Ezh2 has been shown to repress female-biased expression in the male adult mouse liver [81] and is also involved in the regulation of pubertal onset in female rats by inhibiting *Kiss1* expression in the arcuate nucleus prior to the initiation of puberty [82]. Our study highlights that future studies of these mechanisms in the mouse pituitary would be ideal to study prior to puberty.

miRNAs have potentially important roles in responding to sex-biased hormonal release across multiple hypothalamic–pituitary axes. For example, multiple miRNAs have been shown to be estrogen-responsive in the neonatal mouse hypothalamus by the administration of an aromatase inhibitor [26]. miR-1948 and miR-802, identified as sex-biased in adult mouse liver, are known to be regulated by sex-biased pattern of growth hormone release from the pituitary gland [25]. Here we identified two male-biased miRNAs, miR-181-5p and miR-224-5p with estrogen-responsive predicted targets: protein kinase C​​ε (encoded by *Prkce*) whose signaling in response to epinephrine-induced inflammatory pain was found to be suppressed by estrogen in rats [83] and *Ammecr* and *Ank1* whose gene expression is upregulated by estradiol in mouse pituitary [84]. We also identified a female-biased novel miRNA at PD12, novel46, whose host gene, *Cacna1g*, has been shown in anterior pituitary primary cells to promote LH secretion by estradiol signaling through estrogen receptor 1 (ESR1) upon GnRH-induction [84]. While additional functional studies of these miRNAs are needed, these putative miRNA–mRNA connections represent potential gene regulatory networks underlying sex differences in postnatal pituitary gland development.

Sex differences in the number of somatotropes and lactotropes in the adult pituitary have been known for more than two decades [74]. These findings have recently been expanded on by single-cell genomic analyses of the adult pituitary [14–16]. In this study, we combined our temporal bulk RNA-seq data with snRNA-seq data from adult pituitaries to estimate sex differences in cell-type proportions during postnatal development. We suggest that these sex differences in cell proportions occur prior to puberty. It was previously demonstrated that the gonadotrope population displays plasticity in response to reproductive processes, for example, the density of gonadotropes increases in post-pubertal female mice (8–15 weeks) relative to pre-pubertal ages (3 weeks) and the localization of gonadotropes change in lactating mice [85]. In addition, neonatal exposure to testosterone is known to influence the number of somatotropes and lactotropes in the adult male and female rat pituitary [86, 87]. There are several proposed mechanisms by which cell composition changes arise in the pituitary gland: differentiation from the adult pituitary stem cell niche [88], transdifferentiation from differentiated cell-types [15, 89], and self-proliferation from existing cell-types [90, 91]. Future work such as single-cell multiomic profiling of female and male pituitaries during early postnatal development will be needed to pinpoint the gene regulatory networks that govern pituitary cell type composition.

By using bulk mRNA and miRNA profiling of multiple replicates of male and female mice at multiple postnatal timepoints, we identified sex biases in gene expression which are not explained by differences in cell type proportion. Several of these genes have plausible links to regulating sex biases in specific pituitary cell types. For example, the male-biased expression of *Rab3b* in lactotropes is consistent with its inhibitory role of PRL secretion in males [92]. In addition, the male-biased expression of *Gata2* in gonadotropes is consistent with male-specific growth deficiency observed with pituitary-specific Gata2 KO mice [93] and the male-specific reduction in serum FSH in gonadotrope-specific Gata2 KO mice [94]. Our analysis also reveals potential novel sex-biased roles for genes. For example, *Nr4a1* may be female-biased in gonadotropes, and this gene has previously been shown to be upregulated in response to GnRH [95].

Early evidence of the power of single-cell genomics for studying gene regulatory networks active in postnatal pituitary comes from Ruf-Zamojski et al. who used snRNA-seq and snATAC-seq to characterize the sex-biased specific regulatory landscape of the male and female adult mouse pituitary (10–12 weeks) [16]. Particularly, Ruf-Zamojski et al. highlighted a latent variable (LV) showing increased expression/accessibility in females. We found that 10 of the top 30 genes associated with this LV also show evidence of female-biased expression in our study (*Ankra2*, *Crhbp*, *Ddx21*, *Ern1*, *Gadd45g*, *Greb1*, *Npr2*, *Nrg4*, *Rps6ka2*, *Stat5a*). Technological advances in single-cell small RNA sequencing (reviewed in [96]), and in particular single-cell miRNA–mRNA co-sequencing techniques [97], will likely permit a higher-throughput way to assign miRNAs to specific cell types and study relationships with their target genes.

### Perspectives and significance

We performed comprehensive profiling of pituitary gene and miRNA expression across the pubertal transition, a major postnatal developmental milestone, in both male and female mice. We have made this resource freely available so that others can use this data to design experiments to further understand the biology of sex differences in the pituitary gland. Our initial analysis of these data identified novel pituitary-expressed sex-biased genes and miRNAs that cannot be explained by differences in cell-type proportions alone. We also revealed dramatic developmental changes between PD12 and PD22, the mechanisms of which remain to be identified. By providing evidence for pre-pubertal sex differences in pituitary cell-type proportions, another specific challenge is dissecting the mechanisms which generate these differences. The unique role of the pituitary gland in reproductive and stress-related processes that differ between sexes highlights the importance of such future work.

## Supplementary Information

**Additional file 1: Figure S1.** Illustration of 3’UTR-seq method and quality control. A. Overview of the QuantSeq data analysis pipeline. B. Genome browser screenshot of extended 3’UTRs for genes Pou1f1 (left) and Ghrhr (right). Gene name and gene model are shown on the bottom of each panel. Each track represents overlapping signal from 5-6 biological replicates. C. Summary of qPCR vs. 3’UTR-seq comparisons across samples in all samples. Bottom panel: bar plots showing the numbers of genes detected using qPCR (white bars) and 3’UTR seq (red bars). The Spearman correlation coefficients between two experiments are shown for each sample in the dot plot (middle panel) and as well as in a density plot (top panel). **Figure S2.** Correlation heatmaps for pituitary gland samples. Pearson correlation between samples is calculated based on (A) gene expression and (B) miRNA expression. Hierarchical clustering is performed based on pairwise Pearson correlation coefficients (PCCs). Heatmap color intensity represents PCCs (orange: lower correlation; purple: higher correlation). Sample conditions are shown in top bars. Green shades: different ages, blue: male samples; red: female samples. **Figure S3.** Characterization of sex-biased mRNAs and miRNAs. A. Gene expression heatmap of sex-biased genes at PD12 and PD22, and genes with significant sex-by-age effect between PD12 and PD22. Each row represents a gene and each column represents a sample. Column annotation bars indicate sample age and sex. Colors represent row-scaled log2 (normalized counts). Whether a gene is female-biased (red) or male-biased (blue) at each corresponding age is summarized by the row annotation bars on the left. Sex chromosome-linked genes are labeled with asterisks. Genes with significant sex-by-age interaction effect between PD12 and PD22 are bolded. B. Expression plots of all sex-biased miRNAs. Log2(normCounts) are plotted for each miRNA across ages. Large filled points represent median expression at each age and unfilled points represent each biological replicate. Red: female samples; blue: male samples. C. novel46 is a mirtron of Cacna1g. The precursor of novel46 is expressed from the last intron of Cacna1g (highlighted in blue). The canonical seed region (nucleotide positions 2-8) in the mature sequence of novel46 is bolded. **Figure S4.** Summary of gene co-expression modules. Left panel. Module expression profile (log2-transformed normalized counts (log2(normCounts)) scaled per gene across all samples) is plotted for genes within each co-expression module across profiled postnatal ages. Dotted lines: individual gene profiles, solid line: median expression profile of module genes. Number of genes within each module is labeled at the top of the plots. Red: female samples; blue: male samples. Right panel. Expression profile of top five hub genes for each module. log2(normCounts) is plotted across profiled postnatal ages. Large, filled points represent median expression at each age and unfilled points represent each biological replicate. Blue: male samples; red: female samples. **Figure S5.** Characterization of co-expression gene modules. A. Correlation heatmap based on module eigengene (first PC, obtained using “mod_summary()” function from “CEMitool”). Color scale represents pairwise Pearson correlation coefficients. B. Barplots showing the eigengene for each sample in each module. Samples are grouped by age and colored by sex (blue: male; red: female). C. Barplots showing pathway enrichment results for genes in each module. Each bar represents a pathway. Color represents pathway categories (BP: Biological Process; CC: Cellular Component; MF: Molecular Function). Number of module genes in the pathway is labeled. X axis: -log10(P-value) of each pathway. **Figure S6.** Co-expression module gene enrichment in single-nuclei RNA-seq data. Uniform Manifold Approximation and Projection (UMAP) dimension reduction representation of adult female and male mouse pituitary gland single-nuclei transcriptome integrated by sex from Ruf-Zamojski et al. 2021 (A) colored by cell type and (B) colored by sample sex. Samples derived from snap-frozen pituitaries were first merged between replicates for each sex (n=3/sex). Merged samples were then integrated between sexes. C. Enrichment heatmap of co-expression module genes within cell types. One-sided Kolmogorov–Smirnov (KS) test was performed to test for enrichment of each co-expression module gene within a given cell type compared to all other cell types. Color gradient represents KS test FDR-adjusted P-value for each gene (dark purple: high enrichment; gray: low enrichment). Only genes with FDR ≤ 0.05 in at least one cell type are plotted and only FDR < 0.05 are shown. Each column represents a cell type as labeled at the bottom of the heatmap. Each row represents a co-expression module gene and the genes are grouped by the co-expression module in which the gene was identified (labeled on the right). Breaks were added in the heatmap between co-expression modules. Colored boxes represent the level of significance based on a one-sided hypergeometric test to determine if a group of module genes with KS test FDR ≤ 0.05 was significantly enriched for a given cell type. D. Estimated cell-type proportions by RNA-seq deconvolution using Proportions in Admixture (WGCNA) changes across profiled ages of pituitary cell types without known sex differences in their proportions. Estimated cell-type proportions are plotted across postnatal ages along the x-axis. Large circles and triangles represent the mean cell-type proportion at each age and small circles and triangles represent each biological replicate. Lighter color, solid line, circle points: female samples; dark color, dotted line, triangle points: male samples. Wilcoxon test was performed to compare cell proportions between both sexes at each age (*P<0.05, **P<0.01). See Figure 5B for estimated proportions of cell types with known sex biases.

**Additional file 2: Table S1.** Quality control metrics of 3’UTR-seq and small RNA-seq libraries.

**Additional file 3: Table S2.** Novel miRNAs and sequences.

**Additional file 4: Table S3.** Differential analysis results for gene expression.

**Additional file 5: Table S4.** Differential analysis results for miRNA expression.

**Additional file 6: Table S5.** gProfiler pathway enrichment of sex-biased genes.

**Additional file 7: Table S6.** Correlation of miRNA–gene targets.

**Additional file 8: Table S7.** Genes in co-expression modules.

**Additional file 9: Table S8.** Sex-biased genes identified by scMappR in cell types.

## Data Availability

The datasets generated and analyzed during the current study are available in the ArrayExpress repository under accession numbers: E-MTAB-9459, E-MTAB-9460. 3’UTR-seq bigWig files are available in E-MTAB-9459 for genome browser visualization. All datasets and scripts used to perform analyses in the paper and reproduce figures can be found here: https://github.com/wilsonlabgroup/pituitary_transcriptome_analyses.
